# Pigmented Viral-Associated Conjunctival Carcinoma

**DOI:** 10.1155/2013/783104

**Published:** 2013-05-09

**Authors:** Norman C. Charles, Brian P. Marr, Susan M. Stenson, Khushbakhat R. Mittal

**Affiliations:** ^1^Department of Ophthalmology, New York University Medical Center, 550 First Avenue, New York, NY 10016, USA; ^2^Department of Pathology, New York University Medical Center, 550 First Avenue, New York, NY 10016, USA; ^3^The Ophthalmic Oncology Service, Memorial Sloan-Kettering Cancer Center, New York, NY 10065, USA

## Abstract

A 54-year-old African-American woman developed a pigmented papillary squamous cell carcinoma in the palpebral and bulbar conjunctiva of the right eye in areas that received no sun exposure. In situ hybridization performed on the tumor showed human papilloma virus 16. The left eye showed a pedunculated nonpigmented conjunctival dysplasia. The tumors were extirpated by cryosurgery and topical interferon alfa 2b in the right eye and simple surgical excision in the left eye.

## 1. Introduction

Conjunctival squamous cell carcinoma is usually unilateral, solar-induced, amelanotic, and located at the limbus in the horizontal meridian. We report a case of bilateral lesions in nonsun-exposed areas, one of which was pigmented and contained human papilloma virus (HPV) 16. 

## 2. Case Report

**Figure 1:**
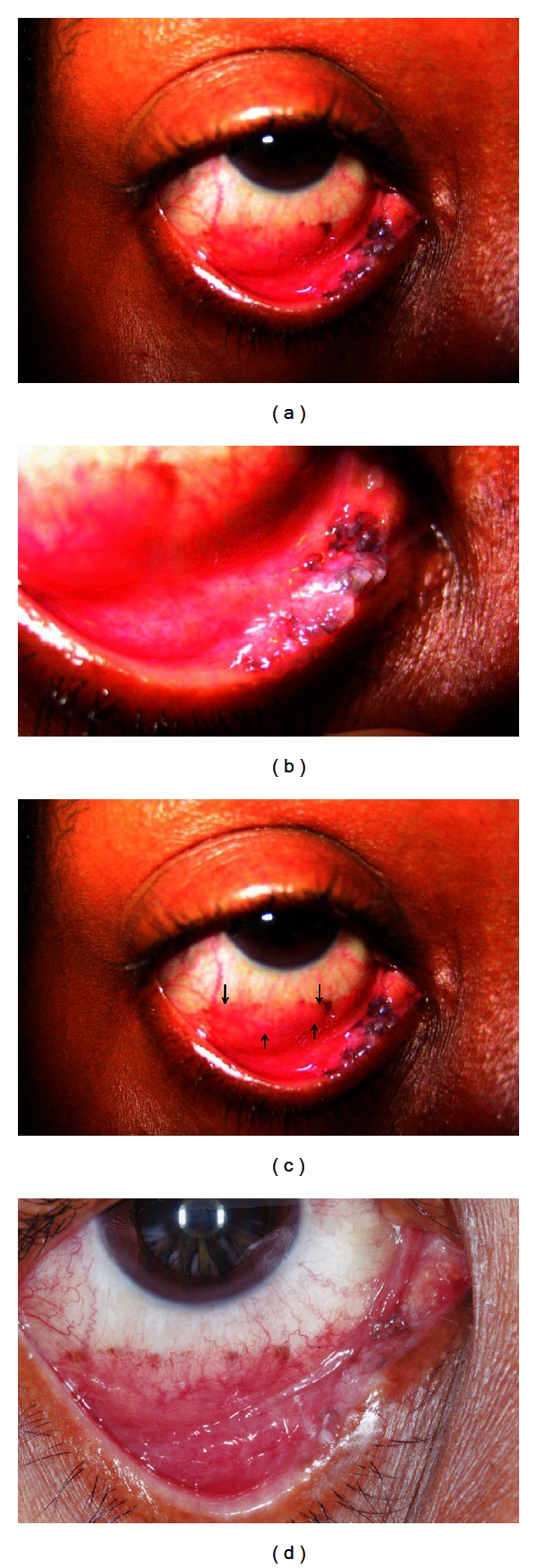
(a)-(b) show partially pigmented papillary tarsal tumor, right lower lid. (c) Region of fine epibulbar papillary tumor (arrows). (d) Tumor has resolved following 2 months of treatment.

 A 54-year-old African-American woman noted an irritation of the right eye of 3-months duration. She was an insulin-dependent diabetic, negative for human immunodeficiency virus (HIV), and a nonsmoker. The right eye showed a gray and brown papillary lesion involving the medial portion of the inferior tarsal conjunctiva (Figure 1). The tumor was 14 mm in horizontal length and spared the lacrimal punctum. A separate ovoid epibulbar plaque of fine, partially pigmented papillae was present on the bulbar conjunctiva inferiorly and measured 17 × 4 mm. There was no preauricular, submandibular, or cervical lymphadenopathy. She wore gas-permeable hard contact lenses for keratoconus with best corrected visual acuity of 20/25 OD and 20/30 OS. There was no other ocular abnormality. Biopsy of the palpebral conjunctiva was performed. 

**Figure 2:**
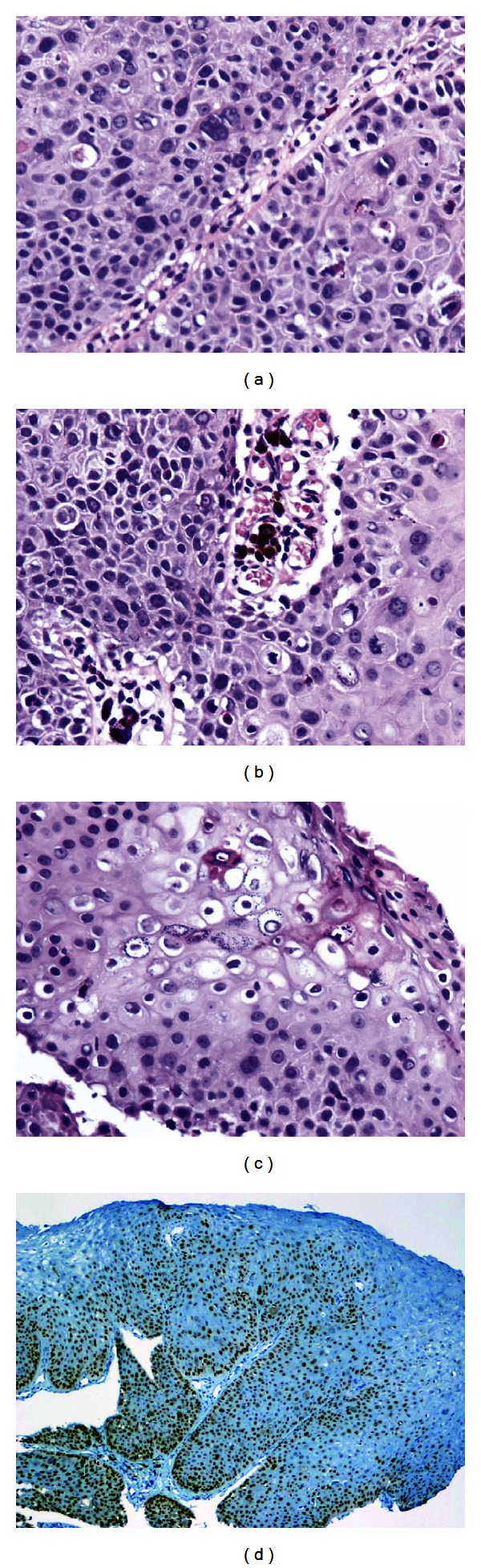
(a) Photomicrographs demonstrate cytologic atypia of tarsal tumor, right lower lid. (b) Pigmented melanophages within vascular cores imparting brown color to tumor. (c) Viral-containing koilocytes are present in outer tumor. (d) P53 immunostain is diffusely positive (hematoxylin/eosin (a)–(c) (×400); (d) immunostain, (×200)).

Pathologic results showed a papillary tumor consisting of mitotically active, pleomorphic epithelial cells with koilocytes within the superficial tumor. Dendritic melanocytes and pigmented macrophages were present in the papillary vascular cores. Immunohistochemical stain for p53 showed positivity in most of the tumor cells (Figure 2). In situ hybridization (ISH) was focally positive for Inform human papillomavirus (HPV) family 16 probe and negative for family 6 probe. 

**Figure 3:**
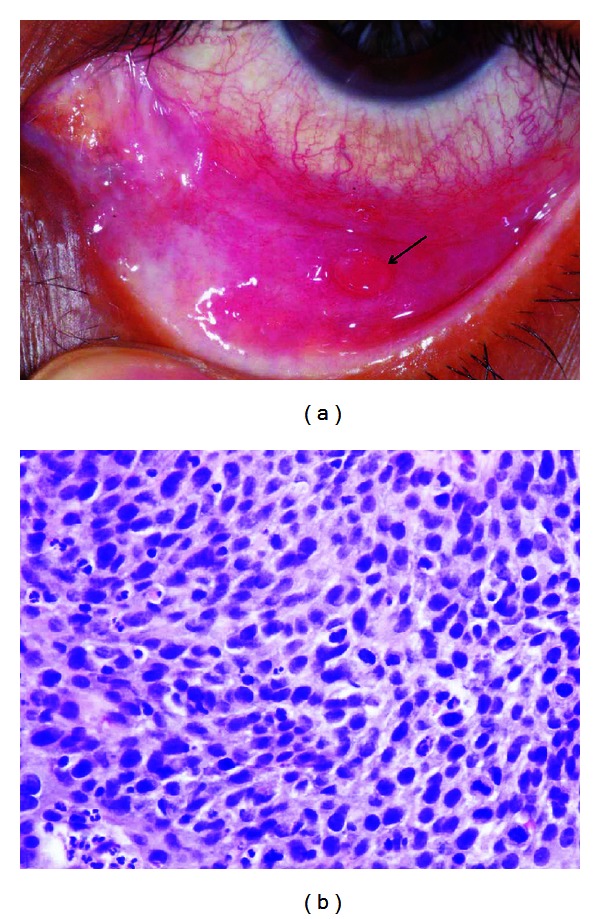
(a) Single pedunculated forniceal tumor is present, left lower lid (arrow). (b) dysplastic tumor cells admixed with inflammatory cells (hematoxylin/eosin (×400)).

 One month later the epibulbar papillae showed continuity with the palpebral lesion. Inferior symblepharon was present. The left eye had developed a pedunculated lesion of the lower fornix (Figure 3(a)). Additional biopsy of the conjunctiva in the right eye disclosed an area of minimally invasive squamous cell carcinoma (SCC). The conjunctival lesion in the left eye was completely excised and proved to be a dysplasia (Figure 3(b)).

The patient underwent cryotherapy in the right eye, followed by interferon alfa 2b topical drops. After a 2-month cycle, the tumor had undergone clinical resolution and showed no recurrence at 8-month followup.

## 3. Discussion

 The principal risk factors for the development of conjunctival intraepithelial neoplasia (CIN) and SCC include sun-exposure with solar elastosis, proximity to the equator, HIV and HPV infection, and increased p53 expression. Cigarette smoking and contact lens wear have also been implicated. The relative importance of these contributing factors is obscured by the fact that they may coexist or overlap in places like equatorial Africa. Studies are also hampered by the relatively small number of cases available for study. Our patient's lesion occurred in a nonlimbal, relatively sun-protected region of the eye but had histologic evidence of viral infection, p53 mutation, and identification by ISH of HPV16 in the right eye.

While it is tempting to extrapolate from the generally accepted causal relation between HPV and uterine cervical carcinoma [1], the etiologic role of HPV in relation to CIN is unclear, with the literature being confusing and contradictory. Suggestive is the fact that DNA from “high risk” HPV types 16 and 18 has been demonstrated in cervical carcinoma as well as ocular CIN, SCC, and papilloma [2]. HPV16 has been demonstrated in each eye in 2 rare cases of bilateral conjunctival dysplasias, in bilateral papillomas [3], and even in normal conjunctiva of patients with genital HPV infection (suggesting autoinoculation). One group studies CIN using reverse transcriptase in situ PCR and detected *active* viral expression of HPV 16 and 18 in mRNA. That report and other proponents of the viral etiology theorize that the protein encoded by the HPV 16/18 E6 or E7 region forms a complex with the protein encoded by the host tumor suppressor gene p53 [4].

 A report of 31 cases of CIN and SCC from Germany, however, found no HPV DNA in the biopsies and concluded that solar elastosis was a major causative factor in these cases, nearly all of which were limbal and papillary [5]. The papillary preponderance is unusual considering that most CIN present with a gelatinous appearance at the limbus. Another study failed to detect HPV 6, 11, 16, or 18 in 20 conjunctival cancers by PCR, but found types 6 and 11 in most papillomas [6]. Similarly no HPV DNA was identified by PCR in a Bangkok study of CIN and SCC [7].

We feel that it is reasonable to infer a spectrum of etiologic factors in conjunctival neoplasia. It will be interesting to determine the effect of recommended pediatric HPV vaccinations on future cervical and conjunctival neoplasia. 

The pigmentation of the lesions in the right eye underscores the fact that both benign and malignant conjunctival epithelial lesions may appear pigmented in dark-skinned patients and may be confused with malignant melanoma [8–11]. In the current case clinical pigmentation reflected melanophages and dendritic melanocytes that occupied the papillary vascular cores. An electron microscopic study of pigmented conjunctival carcinomas [11] identified melanin granules in 4 cell types: neoplastic squamous cells containing a few intracytoplasmic melanosomes, Langerhans cells with occasional mature melanosomes, macrophages containing degenerating melanosomes, and pigmented stellate cells with abundant mature melanosomes within cytoplasmic processes. The latter cells are identical with the cells found in normal human conjunctiva containing racial pigmentation. It is likely that melanocytes migrate from adjacent conjunctiva into the tumor epithelium or the fibrovascular cores of papillary lesions.
